# Clinical validation of the novel CLIA-CA-62 assay efficacy for early-stage breast cancer detection

**DOI:** 10.3389/fonc.2023.1009863

**Published:** 2023-05-03

**Authors:** Marina Sekacheva, Alexander Boroda, Anastasia Fatyanova, Alexander Rozhkov, Nikolai Bagmet

**Affiliations:** ^1^ World-Class Research Center “Digital Biodesign and Personalized Healthcare”, Sechenov First Moscow State Medical University, Moscow, Russia; ^2^ Department of Biliary, Hepatic, and Pancreatic Surgery, B.V. Petrovsky Russian Scientific Center of Surgery, Moscow, Russia

**Keywords:** CLIA, tumor, CA-62, CA 15-3, breast cancer, ductal carcinoma *in-situ* (DCIS), screening, mammography

## Abstract

**Background:**

Without organized screening programs up to 60-70% of breast cancers are diagnosed at advanced stages that have significantly lower five-year survival rate and poorer outcomes, which is a serious global public health problem. The purpose of the blind clinical study was the assessment of the novel *in-vitro* diagnostic chemiluminescent CLIA-CA-62 assay for early-stage breast cancer detection.

**Methods:**

Blind serum samples of 196 BC patients with known TNM staging, 85% with DCIS, Stage I & IIA, and 73 healthy control subjects were analyzed with the CLIA-CA-62 and CA 15-3 ELISA assays. Results were also compared to the pathology findings and to published data from mammography, MRI, ultrasound, and multi-cancer early detection test (MCED).

**Results:**

The CLIA-CA-62 overall sensitivity for BC was 92% (100% for DCIS) at 93% specificity and it decreased in invasive stages (Stage I=97%, Stage II=85% and Stage III=83%). For the CA 15-3 assay sensitivity was 27-46% at 80% specificity. Sensitivity for mammography was 63-80% at 60% specificity, depending on the stage and the parenchymal density.

**Conclusion:**

These results demonstrate that CLIA-CA-62 immunoassay could prove useful as a supplement to current mammography screening and other imaging methods, thus increasing the diagnostic sensitivity in DCIS and Stage I breast cancer detection.

## 1 Introduction

According to current global cancer statistics, female breast cancer has become the most commonly diagnosed cancer globally (more than 2 million estimated cases in 2022 worldwide) surpassing lung cancer (1). The global annual percent change (APC) for BC mortality increased by 0.23% (2). The global deaths from breast cancer increased by 83.95% since 1990 (95% UI: 70.07–96.74%), with 685 000 new cases in 2022 (1). Asia takes a leading place for cancer incidence, followed by Europe and North and South America. For many low-and-middle-income countries from 40% to 70% of breast cancer is detected at advanced stages associated with lower five-year survival rates, which represents a serious global health problem (3). The target population for breast cancer awareness are women over 40 years of age, since breast cancer incidence rate increases with age, hereditary breast, and ovarian cancer syndrome, BRCA2 – germline mutations, and female sex hormones fluctuations from 60 cases per 100,000 in women 30-40 years of age to an average of 430 cases per 100,000 in women 65-75 years of age (4).

The following methods are being used for breast cancer diagnostics, such as bilateral and digital mammography, a conventional ultrasound of the mammary glands for women under 40 years of age, ultrasound elastography, and MRI scanning with contrast. Regardless of all the drawbacks of breast cancer mammography screening of women, such as missing some advanced cancers and producing 3/4 of “suspicious mammograms” associated with benign breast diseases, - mammography continues to be the only proven screening test to decrease mortality from breast cancer (5). At present, only a few countries with a high development index have improved prevention measures and implemented population-oriented breast cancer screening programs as well as improved quality of cancer care, allowing the detection of ~80% of early-stage breast cancer (1). The biggest obstacle to the overall screening approach worldwide is the very high cost of the organized screening programs, which have significant budget implications, depending on the size of the population and the healthcare system resources involved. For instance, the overall costs for annual breast cancer screening of 1,000 women in the general population of Canada are approximately $16.0 million as a lifetime expense (ages 40 to 74) (6). As opposed to the North America and some Western European countries practice, the majority of countries worldwide, including Asia, Arabic, and some Eastern European countries use mammographic screening as an opportunistic diagnostic method since there are no population-based mammographic screening programs (7). To resolve the growing breast cancer problem, it is critical to find an economically viable solution for the prevention of advanced breast malignant disease through early breast cancer detection and optimal access to treatment.

According to the tumor, node, metastasis (TNM) staging classification based on AJCC 8th edition Early-stage breast cancer refers to a malignant neoplasm that has not spread beyond the breast or the axillary lymph nodes (8). This includes Tis – Ductal carcinoma *in situ* or Paget's disease of the breast with no associated tumor mass, Stage I (T1aN0M0, T1bN0M0, and T1cN0M0), Stage IIA (T0N1M0, T1N1M0), and Stage IIB (T2N0M0, T2N1M0, T3N0M0).

For decades cancer biomarkers were extensively used to detect, diagnose, or manage certain types of cancer within the standard of care in many parts of the world. These biomarkers include different types of glycoproteins or various genes known to be associated with cancer, which are formed within the growth of a neoplasm (9). The detection and identification of such cancer biomarkers in a patient’s body fluids provide valuable data in regard to the diagnosis of invasive breast cancers, providing prognostic information and predicting response to a chosen therapy, and a selection of the strategy for treating cancer, which leads to improved outcomes. However, there is a limitation of using circulating biomarkers related to their low sensitivity (10-30%) for detecting early stages of breast cancer (10).

In recent years, our cancer research group has been studying various combinations of biomarkers (CA-125, CA 15-3, CEA, CYFRA 21.1, D-dimer, HE4 etc.) for their effective use in screening for different types of cancers (11, 12), including breast cancer (13). In this regard, we aimed to identify novel emerging biomarkers for their successful use in combination with other well-known cancer markers, which can significantly improve the accuracy of cancer screening using a classification model. Thus, in the last 5 years, our attention has been drawn to various pilot clinical studies that were carried out by our colleagues using a novel marker for epithelial carcinomas CA-62 and the results were presented at various international symposiums.

The purpose of this study was the assessment of the diagnostic characteristics of the novel FSSH-approved (Federal Service for Surveillance in Healthcare of the Russian Federation) *in vitro* diagnostic chemiluminescent immunoassay (IVD CLIA-CA-62) for early-stage breast cancer detection as compared to healthy controls. The same samples were analyzed with FSSH-CA 15-3 ELISA assay, and the results were compared to histopathologic diagnosis used as a gold standard.

The significance of this study is related to the unique qualities of the highly sensitive CA-62 marker, which allows detecting it in the blood of patients with ductal carcinoma *in situ* (DCIS) and Stage I breast cancer present in asymptomatic women. This study has the potential to provide insight into the usefulness of the CA-62 biomarker as a first-line test to select subjects at high risk for developing breast cancer (BC) who need further mammography, potentially avoiding radiological exposure in low-risk BC individuals who test negative.

CA 15-3 ELISA-BEST assay is based on the well-known cancer marker CA 15-3, an O-glycoprotein member of the mucin family commonly used for breast cancer control management (11, 14). It is a protein product of the MUC-1 gene, which is shed into the bloodstream from adenocarcinomas in a reduced glycosylated form. Despite its low sensitivity for early stages of breast cancer detection, CA 15-3 cancer antigen is extensively used for cancer treatment monitoring in combination with clinical examination and various imaging methods and for early detection of cancer recurrence (14, 15).

Human CLIA-CA-62 immunoassay is based on the novel marker for epithelial carcinomas CA-62, which is a carcinoma-specific mesenchymal marker, expressed on the epithelial cell surface of the EMT-transformed undifferentiated stem cells from the onset of cancer development. There are some previous publications describing CA-62, a patented set of reagents CLIA-CA-62 based on the biomarker CA-62 for early cancer detection (16–18) and monitoring response to chemotherapy (19). A marker for epithelial carcinomas CA-62 represents a family of low-weight membrane transport N-glycoproteins that bind alpha-fetoprotein (AFP) using a special combination of the branched polysaccharides, which are located on the mesenchymal cells’ surface and function by Clathrin-mediated endocytosis. Tumor cells release into the blood two main soluble cytoplasmic fractions of N-glycoprotein that are detected by the antibody used in the test. This allows to quantitatively measure a serum level of CA-62 antigen using a specific chemiluminescent assay CLIA-CA-62 intended to help with the medical decision-making process and recommended for early cancer detection in combination with clinical data and other diagnostic procedures (16, 17).

Test performance of both assays was also compared to histopathological findings and to published data for mammography, conventional ultrasound, ultrasound elastography, MRI, and also to blood-based multi-cancer early detection test MCED (from GRAIL Inc.).

## 2 Materials and methods

### 2.1 Study design and participants

#### 2.1.1 Patients

Patients with histopathology-confirmed breast cancer before the treatment (N=57) and healthy control subjects (N=73) were enrolled in an observational clinical study in 2018 at the Institute for Personalized Medicine of the Sechenov First Moscow State Medical University (“Sechenov University”), Moscow, Russia. Inclusion criteria for breast cancer patients: women of any race and ethnicity between the ages of 25 and 80, who have been diagnosed with either DCIS or Stage I, Stage IIA-B, or Stage III of primary breast tumor with or without lymph node metastasis; histopathological confirmation of breast cancer, which was used for definitive diagnosis of the breast disease. Exclusion criteria for this study included factors such as age before 25 and above 80, more advanced stage of disease (Stage IV), and previous treatment history.

Breast tissues were collected from resected breast tumors at the time of mastectomy or lumpectomy, fixed in formaldehyde, and embedded in paraffin. The tissues were cut into sections and stained with hematoxylin/eosin. The diagnosis of a benign or malignant breast tumor was confirmed by certified pathologists. Histopathological classification and staging were performed according to AJCC eighth edition (8).

Healthy control subjects were selected based from a large pool of apparently healthy individuals on matching variables of interest such as age (from 25 – 80), same gender, any race and ethnicity, free of cancer, with normal biochemical and full blood count reference intervals seen in a healthy reference population according to the international standard ISO 15189:2012. Exclusion criteria for healthy control subjects included several factors, such as another gender (men), age before 25 and after 80, presence of comorbidities or verified breast benign disease or breast cancer.

#### 2.1.2 Ethics approval and consent to participate

The study was approved by the Local Ethics Committee of Sechenov First Moscow State Medical University. All patients were given informed consent to participate in the study. In total 57 patients with histopathology-confirmed BC and 73 healthy control subjects were included in the analysis. Serum samples were collected at the Sechenov University Hospital after overnight fasting and delivered to the Clinical laboratory.

#### 2.1.3 Serum samples

The total of 269 blinded serum samples included 196 breast cancer patients with known TNM classification (8, 20) and 73 healthy control subjects. Sera from healthy control subjects (N=73) and pre-treatment breast cancer sera (N=57) were collected at the Sechenov University Hospital after overnight fasting and delivered to the Clinical laboratory, processed and stored at −86°C until they were analyzed for CA-62 and CA 15-3 markers. Another set of archived histopathology-confirmed breast cancer sera (N=139) was obtained from the Biospecimen bank ProMedDX LLC, MA, USA. The entire set of serum samples was separated by centrifugation (1300 g, 10 minutes) in BD SST tubes with silica clot activator, and separating polymer gel, heat-inactivated at 56°C for 30 min using standard operating procedures for serum collection (21, 22), and stored at −86°C until used. Serum samples were collected under an IRB-approved protocol from Federal licensed/registered facility following GMPs. The majority (85%) of cancer samples were from patients with Stage I and Stage II cancers, as well as DCIS (T0). The baseline characteristics of the studied serum samples are presented in **Table 1**.

**Table 1:** Baseline characteristics of the analyzed serum samples.

	Breast cancer (N = 196)	Normal control subjects (N = 73)	Total (N = 269)
Age, years			
Mean (SD) Median Range	61years 63years 25 – 93 years	55 years 54 years 45 –70 years	61 years 62 years 25 – 93 years
Age group, n (%)			
< 50 years 50 – 60 years 61 - 65 years >65 - 69 years >70 – 79 years >80 years	31 (15.8%) 36 (18.4%) 34 (17.4%) 32 (16.3%) 49 (25.0%) 14 (7.1%)	21 (28.8%) 35 (47.9%) 9 (12.3%) 6 (8.2%) 2 (2.7%) 0 (0%)	52 (19.3%) 71 (26.4%) 43 (16.0%) 38 (14.1%) 51 (19.0%) 14 (5.2%)
Race, n (%)			
Caucasian Asian	167 (85.2%) 29 (14.8%)	73 (100%) 0 (0%)	240 (89.2%) 29 (10.8%)
Clinical cancer stage, with TNM classification n (%)			
Stage 0 (DCIS, pT0N0M0) Stage I A, B– (pT1N0M0) Stage IIA - (pT1-2N0-1M0, Stage II B - (pT2-3N0-1M0 Stage III A – (pT2-3N1M0) Stage III B – (pT1-4N2M0) Stage IV – (pT0-NxM1)	14 (7.1%) 98 (50.0%) 56 (28.6%) 17 (8,67%) 4 (2.04%) 7 (3.57%) 0 (0%)	none	
Region, n (%)			
USA Russian Federation	139 (70.9%) 57 (29%)	0 (0%) 73 (100%)	139 (51.7%) 130 (49.3%)
Method of cancer diagnosis, n (%)			
Identified by screening test Identified by clinical presentation	139 (70.9%) 57 (29%)	0 (0%) 73 (100%)	139 (51.7%) 130 (49.3%)

#### 2.1.4 Study design

**Figure 1:**
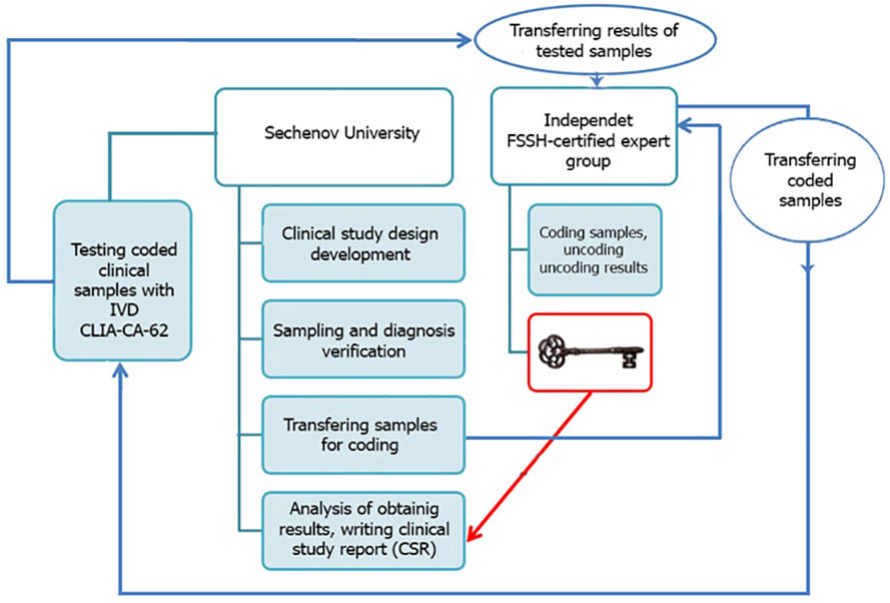
A clinical study methodology.

The entire blind set of serum samples (N=269) was analyzed using an FSSH-approved IVD medical device CLIA-CA-62 based on a competitive chemiluminescent assay and 177 BC cases together with 73 healthy control subjects were tested by another FSSH-approved IVD medical device ELISA-CA15-3 based on a sandwich enzyme-based immunoassay. A controlled blind clinical study using serum samples from histopathologically confirmed patients was carried out at Sechenov University, Moscow. A clinical study methodology is presented in **Figure 1**. The blinding was carried out by experts from the external independent laboratory of the Federal Service for Surveillance in Healthcare of the Russian Federation. Such study design provides a high level of internal validity and allows avoiding any bias, chance or confusion.

### 2.2 Human CLIA-CA-62 immunoassay

Measurements of CA-62 cancer antigen in patients’ sera were performed using the *in vitro* diagnostic immunoassay from JVS Diagnostics LLC, Moscow, Russia (Lot# CLIA-CA-62-200221), according to the manufacturer’s instruction. IVD CLIA-CA-62 chemiluminescent immunoassay approved by the Federal Service for Surveillance in Healthcare of the Russian Federation (FSSH) is intended for the quantitative measurement of carcinoma-specific antigen CA-62 in human blood serum. A set of reagents CLIA-CA-62 is a one-step solid-phase competitive chemiluminescent immunoassay, in which a competition takes place between the carcinoma-specific antigen CA-62 contained in the test sample and the labeled cancer antigen CA-62-Acridinium (Acridinium NHS-ester) for binding to monoclonal antibodies (Mabs) to glycoprotein CA-62, immobilized on the solid phase (96-well plate) (18). The human CLIA-CA-62 test kit contains all the required sets of reagents to analyze 48 samples in duplicates, including the CA-62 standard calibrators. During 2-hour incubation, a solution containing a diluted serum sample (1:5), 50 µl of Positive control sample, 50 µl of Standard Calibrators CA-62, and 50 µl of the labeled cancer antigen 400 ng/ml solution were added to the wells with immobilized antibodies, thereafter the sorbent was washed away from unbound components. A series of standard calibrators CA-62 were tested simultaneously with the specimens to plot a Logit-Log calibration curve for the quantitative determination of the glycoprotein CA-62 in measurement units (U/ml) of the unknown samples. The measurable luminescent signals from the immune complexes {anti-CA62-Mab-(CA-62-Acridinium ester)} formed on the solid phase were then recorded immediately after the injection of the activating buffer solution (integration time 0.3 sec) in the wells, which induces the photon emission, detected by a flash chemiluminescence reader. The intensity of the luminescent signal is inversely proportional to the concentration of the measured analyte in the specimen. Samples with CA-62 units higher than the largest CA-62 calibrator were diluted accordingly with a working buffer followed by the determination of the exact concentration by multiplying on the dilution factor.

Measurements were made following the manufacturer’s instructions. Sensitivity of the assay: 35 U/ml; the assays had linearity of 91 and 105% over the measurement range of 1250 -10000 U/ml. The total analysis time was 4 hours. Detection method: flash chemiluminescence using Tecan Spark (Tecan Trading AG, Switzerland, EU). The cut-off value (5000 U/ml) used was recommended by the manufacturer based on CA-62 levels in sera from 353 healthy individuals 18 – 65 years old. All the test samples were done in duplicates using Standard calibrators CA-62 and Positive control samples as reference standards included in the set of reagents. 95% of the sera from healthy control subjects were above the limit of detection (LOD). The intra-assay coefficient of variation was ≤ 10%, over the range of concentrations.

### 2.3 The sandwich CA 15-3 ELISA

Measurements of CA 15-3 cancer antigen in studied serum samples were performed using a solid-phase sandwich CA 15-3 ELISA-BEST (FSSH-approved IVD medical device from Vector-BEST, Novosibirsk, Russia, Lot#T-8472) in accordance with the manufacturer’s instruction. The CA 15-3-ELISA-BEST is designed to quantitatively measure the amount of cancer antigen CA 15-3 bound between a matched antibody pair in human serum. It uses two types of monoclonal antibodies specific to different CA 15-3 epitopes. Capture CA 15-3 specific monoclonal antibodies have been pre-coated in the wells of the supplied microplate. Samples, CA 15-3 standards, positive controls, and substrate solution for the secondary peroxidase HRP- labeled antibody are then added into the corresponding wells, allowed to react with the HRP-antibody-CA-15.3 complex to produce a measurable optical signal to be recorded with a colorimetric microplate reader. The sandwich is formed by the addition of the secondary antibody. In this case, the intensity of this signal is directly proportional to the concentration of CA 15-3 antigen present in the original specimen. This approach to sandwich ELISA allows the formation of the antibody-analyte sandwich complex in a single step. Time-to-result: 3.5 Hrs. Sensitivity of the assay: 0.5 U/ml, diagnostic range: 10 U/ml - 250 U/ml, detection method: colorimetric. For the optical density measurements was used a Tecan Spark (Tecan, Switzerland, EU). The assay had linearity ranges from 97 to 98% over the range of concentrations from 5 to 65 U/ml. The cut-off value (30 U/ml) was recommended by the manufacturer based on CA 15-3 levels in sera from healthy females (N=97) of 18 – 50 years old. All the test samples were done in duplicates using Standard calibrators CA 15-3 and Positive control sample as reference standards included in the set of reagents. The intra-assay coefficient of variation was ≤ 8.2%.

### 2.4 Statistical analysis

The distribution of CA-62 and CA 15-3 in sera from healthy and breast cancer patients was tested for normality using the D’Agostino-Pearson omnibus test. The Pearson correlation coefficient (r) was used to determine the correlation between CA-62 and CA 15-3 serum levels. Since CA-62 values a 1000 times higher than the CA 15-3, the original values for both cancer markers in different subgroups were log-transformed (log10) before the analysis for obtaining the same equivalent scales, which allow getting a graphical correlation with y = a*Lg(x) + b. For the evaluation of the difference between cancer and healthy control groups the Mann–Whitney U test was used. In order to evaluate the diagnostic characteristics of each cancer antigen we calculated the sensitivity and specificity, test accuracy, PPV, and NPV, and compared the cancer samples to normal control subjects using the receiver operating characteristic (ROC) analysis for the two markers. The level of significance was set at p < 0.001. Statistical analyses were performed using the MedCalc statistical software (version 19.7.4, MedCalc Software Ltd, Belgium, EU). The weighted kappa k-coefficients were used for evaluation of the diagnostic test results against a gold standard, which is, in our case, the results of the histopathological findings.

## 3 Results

**Figure 2:**
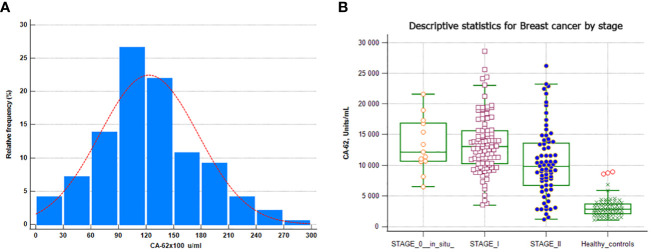
CA-62 normal distribution in breast cancer samples. **(A)** CA-62 levels in sera from healthy controls and from all breast cancer groups **(B)** Error bars denote maximum and minimum values.

Serum samples from 73 healthy control subjects (women) and 196 patients with histopathologically confirmed breast cancer were analyzed for CA-62 cancer marker and 177/196 (due to insignificant sample’s volume) of breast cancer samples with 73 healthy control subjects for CA-15-3 serum levels, as described in the Materials and methods section. The values obtained are shown in **Table 2**. Significantly higher serum CA-62 levels were found in sera from breast cancer patients compared to healthy control women, and the glycoprotein concentration ranged from 1178 to 28598 U/ml (mean ± SD=12312 ± 5326) (**Figure 2**). The median CA-62 values were very high in all stages of breast cancer: ductal carcinoma *in situ* DCIS (12133 U/ml), Stage I (13045 U/ml), Stage II (9824 U/ml) and Stage III (17247 U/ml) as compared to healthy control subjects (2821 U/ml). Interestingly enough, the CA-62 detection level decreases with the tumor stage and demonstrates a very significant production of the marker for epithelial carcinomas from the onset of carcinogenesis, when cancer stem cells are poorly differentiated.

**Table 2:** Diagnostic methods comparison: CA 15-3 ELISA, CA-62 CLIA, mammography, MRI, UE, and MCED in relation to clinical and pathologic data of breast cancer patients.

Parameter	CA-62, U/ml Patient no (% > 5000)	CA15-3, U/ml Patient no (% > 30)	Mammograph ^[29, 36]^ vs DM, Patients % with positive results	MRI FAST^[35]^ Patients % with positive results	UE Patients % with positive results	cfDNA based Multi-cancer early detection test MCED^[30]^
Stage DCIS (0)	14/14 (100%)	2/11 (18%)	36 vs 41%	80%	N/A	N/A
Stage I	95/98 (97%)	38/88(43%)	34% vs 46.6%			16.8%
Stage II	61/73 (85%)	37/68 (54%)	80% - 90%			40.4%
Stage III	9/11 (83%)	5/10 (50%)	80% vs 92%			77%
Stage IV			92%			90.1%
Total	180/196(92%)	82/177(46%)	63 vs 90%	64.8%	80%	51.5%
Sensitivity %	92%	42%	63-90% vs 97%	77 - 80%	81%	51.5%
Specificity %	93%	92%	60% vs 64.5%	64.2%	78.5%	99.5%
AUC	0.955	0.77	78% vs 89%	0.79	0.867	44.4%
PPV	97.8%	93%	80% vs 90.9%	N/A	75%	99.4%
NPV	81%	37%	75% vs 89.3%	N/A	85%	N/A
Test Accuracy%	92.2%	58%	83% vs 89%	75.5%	80.5%	N/A

*Published data based on accomplished screening programs.*Using 0.7 breast cancer prevalence (USA and Western Europe) for population-based screening.

The CA 15-3 values in the breast cancer sera showed a broad range, from 0 to 330.4 U/ml with a median of 25.8 U/ml (mean ± SD= 35 ± 35.6) with its minimum (20.5 U/ml) at Stage I and its maximum (38.2 U/ml) at Stage III. A LOD value of 5 U/ml for the CA 15-3 ELISA immunoassay was used. The entire set of sera from healthy women had CA 15-3 antigen levels above the detection limit (5 U/ml) and 97% of them had detectable CA 15-3 levels in the range of 5 to 65 U/ml (the estimated mean ± SD = 0.2 ± 0.05). Less than half of women (46%) with confirmed breast cancer had elevated levels of CA 15-3 as compared to healthy control subjects. D’Agostino-Pearson omnibus normality test for CA-62 levels in healthy women as well as in breast cancer patients showed Gaussian normal distributions, whereas cancer antigen CA 15-3 levels were not normally distributed for breast cancer patients as compared to healthy control subjects. Both CA-62 and CA 15-3 glycoprotein levels in healthy as well as in breast cancer patients did not have any significant correlation with the age of the individuals at 95% Confidence interval (r = 0.129, p = 0.07 for CA-62 and r = 0.11, p = 0.16). No correlation was found between the serum CA-62 and CA 15-3 levels in the healthy control group and in breast cancer patients (r = 0.11, p < 0.0003). Overall, a significant correlation was found between the CLIA-CA-62 assay and the histopathological findings (r = 0.942, p < 0.0002) using linear regression for the entire set of breast cancer and r = 0.97, p < 0.0001 for Stage I.

**Figure 3:**
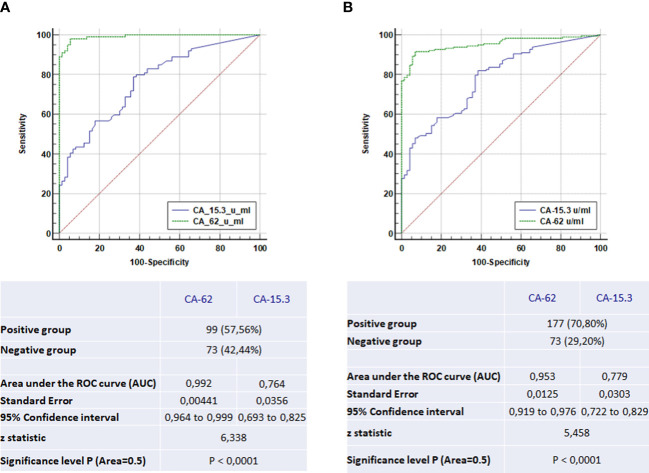
The ROC-curve comparison of the CLIA-CA-62 and CA 15-3 ELISA IVD assays for DCIS and Stage I breast cancer patients in comparison with healthy controls **(A)** and the ROC-curves comparison of the CLIA-CA-62 and CA 15-3 ELISA assays for all stages of breast cancer **(B)**.

The overall performance of the competitive CLIA-CA-62 and CA 15-3 ELISA assays in sera from breast cancer patients with stages I-III and the ductal carcinomas *in situ* was evaluated by constructing ROC curves. The results of the ROC-curve analysis for Stage I and DCIS for both CA-62 and CA 15-3 are shown in **Table 2** and **Figure 3A** and the ROC-curves for all stages are presented in **Figure 3B**. ROC curve analysis for the entire set of breast cancer samples using the CA-62 cancer marker demonstrated a very high AUC = 0.955 with p < 0.001. The CLIA-CA-62 assay for DCIS and Stage I breast cancer showed an AUC of 0.989 with p < 0.0001 using a cut-off value of 5000 U/ml recommended by the manufacturer of the assay; Sensitivity was 97% at 95% specificity (**Figure 3A**) with the median and an average equal to 12133 and 13062, correspondingly. By contrast, the CA 15-3 ELISA assay yielded an AUC = 0.779, p < 0.001 for the entire set of samples, and using a cut-off value of 30 U/ml, the Sensitivity was 46% at 93% Specificity, which corresponds well with previously published studies. The ROC-curve analysis of CA 15-3 ELISA assay for DCIS and Stage I breast cancer showed an AUC of 0.76, p < 0.0001 with a Sensitivity of 40% at the same 93% Specificity (**Table 2**; **Figure 3**). The comparison of the results between the two cancer markers revealed that the Sensitivity of the CLIA CA-62 chemiluminescent assay was approximately double that of CA 15-3 ELISA for early stages of breast cancer, and over three times as high in DCIS (27% vs. 100%). The accuracy of the test (the proportion of the correct test results in a total number of cases) among all examined patients using the CLIA-CA-62 assay is 97% for detecting Stage I breast cancer compared to 92% for the entire set, whereas for sandwich CA 15-3 ELISA it is only 40% (46% for the entire set). The positive predictive value (PPV) and negative predictive value (NPV) were used to describe the performance of the diagnostic test. Further analysis compared the CA 15-3 and serum CA-62 glycoprotein values with published results for other methods of cancer diagnostics such as mammography, ultrasound, and MRI. The Positive predictive value (PPV) for CLIA-CA-62 is 97.8% as compared to 93% for CA 15-3, 78% - 90% for mammography and 75% for MRI and ultrasound. At the same time, test accuracy is the highest at 92.2% for CLIA-CA-62 as compared to 58% for CA 15-3 ELISA, and 75-85% for mammography, ultrasound, and MRI.

## 4 Discussion

This study is unique in terms of the sensitivity demonstrated for the detection of the very early stages of breast cancer in asymptomatic women.

The aim of this study was not a differential diagnosis between breast cancer and breast benign disease using a biomarker CA-62 or an evaluation of the relationship between the level of CA-62 and different molecular subtypes. The main goal was to independently evaluate the diagnostic characteristics of the novel CLIA-CA-62 assay for early stages of breast cancer detection as compared to other CA 15-3 ELISA based on well-known cancer marker CA 15-3 and its prospective use thereof. However, the obtained results have demonstrated a potential in the future to carry out a prospective clinical study of the relationship between the CA-62 serum level and the tumor grade, as well as the molecular subtypes of breast cancer patients.

Measuring serum levels of cancer markers CA-62 and CA 15-3 in 269 samples has established that CA-62 antigen was increased in 180/196 (92%) (p < 0.0001) of breast cancer patients in the DCIS, Stage I, Stage II, and Stage III, whereas elevated CA 15-3 values were found only in 46% (82/177). Both cancer markers in the healthy control subjects’ group (N=73) had serum levels below the upper limit of the reference range (67/73 for CA-62 and 164/177 for CA 15-3). Cut-off values for CA 15-3 ELISA assay were > 30 U/ml, and > 5000 U/ml for CLIA-CA-62 upon which the sensitivity, specificity, AUC, and CI were calculated. Interestingly enough, patients with Stage I of breast cancer and non-invasive ductal carcinoma *in situ* DCIS have demonstrated from 97 to 100% detection using the novel CLIA-CA-62 assay, when curability is the highest, while the mucin-based CA 15-3 ELISA assay was not (27% detection only for DCIS). The same trend was observed for the entire set of breast cancer samples. Previous studies on cancer biomarkers for breast cancer (15) have demonstrated that their low sensitivity and specificity prevent from the use of serum markers such as the MUC-1 mucin glycoproteins (CA 15.3, BR 27.29) and carcinoembryonic antigen (CEA) for the diagnosis of early breast cancer. At the same time, serial measurement of these markers can result in the early detection of recurrent disease as well as reflect the efficacy of therapy.

The reason for such unusually high Sensitivity for early stages of BC detection found in this blind study could be that N-glycoprotein CA-62 is a mesenchymal light N-oligosaccharide, which is shed into the bloodstream far beyond the other cancer maker production. In general, epithelial tumors in a process of malignant transformation gradually lose their differentiation due to the destruction of the connections with the tumor microenvironment, which is controlling the degree of cell differentiation loss, up to the epithelial-mesenchymal transition with the formation of the tumor stem cells and re-expression of the embryonic antigens. During EMT epithelial cells lose their epithelial characteristics, and their polarity and gain some properties of the mesenchymal cells, such as spindle shape, anterior-posterior polarization, and strong migratory potential and mesenchymal markers. As a result, various epithelial cells possessing different morphological and phylogenetic classifications are transformed into the same pluripotent cancer stem cells. From the onset of carcinogenesis heavily branched N-glycoproteins are expressed in large quantities on the cell membrane of such “transformed” stem cells and act as a carcinoma-specific N-glycoprotein MEC/CA-62 detected by the CLIA-CA-62 chemiluminescent assay.

Authors (16, 17) revealed that a CA-62 marker associated with epithelial tumors can be significantly expressed and detected to varying degrees in the tissues of malignant tumors (such as breast, prostate, lung, uterus, stomach, kidney, colon, and ovaries), as well as in various human biological fluids (including blood and saliva). At the same time, healthy control subjects do not demonstrate increased expression of the CA-62 marker. However, some breast benign specimens (<10%) have demonstrated a slight increase in CA-62 level that might indicate a transitional stage of the tumor becoming malignant, which was actually confirmed lately for some benign patients (18). Patients having a strong elevation in serum CA-62 level might have another type of carcinoma, which does not make it false positive for breast cancer detection, but rather a substantial reason for simultaneous detection of other existing cancer.

As compared to low-weight CA-62 N-glycoprotein, a majority of other cancer markers including CA 15-3 represents heavyweight O-mucins (up to 800 kDa), are getting produced when the cancer cells differentiation reaches maturity and are released into the blood after the tumor cells destruction. In this case, the level of released into the blood accumulated tumor-specific and tumor-associated markers is proportional to the tumor growth. That could be a reason for the low detection level of CA 15-3 in the serum of patients with non-invasive or micro-invasive breast lesions as compared to amounts seen in sera from patients with advanced cancers (9, 15). The accepted overall established sensitivity of the CA 15-3 assay for breast cancer detection is in the 20 to 50% range, which is in agreement with the findings reported herein (Se = 27 - 56%) with lower values for DCIS (27%) and Stage I (42%), and higher values for Stage II (54%) and Stage III (50%) (23, 24).

According to the ASCO guidelines, CA 15-3 and CEA can be used only together with physical examination and imaging (23, 24), but mammography has a limited sensitivity of 63-90% due to many possible influences such as unclear lesions, poor aligning, dense parenchyma, calcifications, distortions, and misinterpretations (25). Hence, the probability that a patient with breast cancer will be detected by mammography alone is only 70-90%, and therefore the probability that it will be missed is 10 – 30%. Some multicenter trials established that up to 40% (30% at Stage I) of breast cancer cases are “missed cancers” due to detection and interpretation errors. At the same time, mammographic screening detects 50 per 100,000 (0.5%) invasive breast cancers in women while generating 2200 per 100,000 women (2.2%) false positive results from 90-99% of true negatives (26, 27).

Traditional bilateral mammography is being improved by using contrast-enhanced digital mammography (CEDM) which allows visualizing neovascularization associated with angiogenesis. Other greatly demanded and valuable screening methods are breast ultrasound and ultrasound elastography (UE), which shows superior advantages in differentiating benign and malignant breast tumors as compared to conventional ultrasound (28). On the other hand, breast MRI has some advantages over mammography since it does not use radiation, and is faster and exceptionally safe. MRI images reflect the tumor’s molecular and genetic characteristics. Unlike mammography, which generates images based on the density of the tissue, MRI has higher sensitivity by creating a “blood flow map” which allows visualizing tumor neovascularity, associated with some metabolic modifications that correlate with the proliferation and metastatic potential of the tumor (29, 30).

The published results for various methods of cancer diagnostics such as mammography (MMG), magnetic resonance imaging (MRI), an ultrasound and ultrasound elastography (UE), and DNA-based Multi-cancer early detection test MCED (31) were compared with results obtained for 196 patients with breast cancer using immunological methods CLIA-CA-62 assay and CA 15-3 ELISA. A weighted kappa-test (k) was performed to evaluate the diagnostic consistency of the mammography, CA 15-3 ELISA and CLIA-CA-62 assays with the results of histopathology. The sensitivity and specificity values for the combination of the instrumental methods of diagnostics, such as UE & MRI were 95.8% and 92.8%, correspondingly, which are comparable to the values obtained with the CLIA-CA-62 assay. Kappa coefficients are being interpreted as indices of the test quality in evidence-based medicine (32). In this case, the values of the weighed kappa-coefficient demonstrate a significant difference between the two diagnostic methods: 0.63 (UE&MRI) vs 0.80 (CLIA-CA-62). The comparison of the kappa coefficient (0.8) for the CLIA-CA-62 test with published elsewhere (28) kappa coefficients for UE, MRI, UE&MRI (0.512, 0.527 and 0.630, respectively, p<0.001), for mammography (0.52), and CA 15-3 ELISA (0.22) with significance level p<0.0001 indicate that the CLIA-CA-62 test classifies patients more reliably due to the lesser likelihood of a random coincidence of the test results with the histopathological findings.

Taken together, the results obtained in this independent double-blind study clearly demonstrate that the novel CLIA-CA-62 chemiluminescent assay has significant diagnostic advantages in detecting early stages of breast cancer as compared to other imaging diagnostic methods as well as to the other cancer markers including CA 15-3. The values of sensitivity, specificity, and accuracy of the CLIA-CA-62 IVD assay were 92%, 93%, and 92.2%, which is approximately 1.5 times higher as compared to various visual methods of diagnostics such as MRI, mammography, ultrasound etc. It is especially worth emphasizing the significant difference in detection of DCIS and Stage I & II. When using the CLIA-CA-62 assay, the detection rate was over 97%, whereas the other methods show a range of values from 27% for the CA15-3 assay (23, 24), to 55% for multi-cancer early detection test MCED (31) to 80% for FAST MRI (29).

The comparison of different breast cancer diagnostic methods allows concluding that only a combination of several methods is superior to the single use of either method for the detection of Stage I breast cancer. Data from **Table 2** confirms that a combination of the CLIA-CA-62 and other methods of diagnostics including mammography could significantly improve the detection of non-invasive DCIS and Stage I breast cancer both having a high survival rate.

In the future, it seems appropriate to conduct a clinical approbation on a breast cancer screening of a group of women patients to develop a working algorithm for reliable detection of early-stage breast cancer using the CLIA-CA-62 immunoassay as a pre-screening tool before or in conjunction with breast mammography. It could be beneficial for current breast cancer screening algorithms and particularly for early-stage breast cancer detection. Another clinical study aiming the use of the CLIA-CA-62 immunoassay for differential diagnosis in women above 40 years old with BI-RADS 2, 3, and 4 mammograms, with moderate to high suspicion of breast cancer correlating mammographic and the CLIA-CA-62 data with pathologic findings might help with the interpretation of the pathologic findings and with the differentiation between benign lesions and malignant neoplasms.

## Conclusions

1. The CLIA-CA-62 assay demonstrated 100% sensitivity at 93% specificity for DCIS and 97.8% for Stage I breast cancer as compared to another known cancer marker for breast cancer, such as CA 15-3 (Se = 46%, Sp = 93%) and mammography (Se = 63-80% and 60% Specificity depending on the stage of cancer and parenchyma density).
2. Cancer marker CA-62 has a few unique qualities that distinguish it from other well-known cancer markers: it is present at a very high level in the blood of patients (>97%) with asymptomatic ductal carcinoma *in situ and* Stage I breast cancer and it doesn’t increase along with the cancer progression and differentiation. Since the carcinoma-specific marker CA-62 appears on the surface of the transformed mesenchymal epithelial cancer cells in the course of carcinogenesis, it is gradually fading away, when tissue differentiation reaches maturity.
3. There is a significant level of agreement (k=0.8) between the CLIA-CA-62 assay results based on the marker for epithelial carcinomas CA-62 with histopathological findings for the entire set of breast cancer, including Stage I.
4. Despite the fact that the CA-62 is a marker for epithelial carcinomas and is not specific for breast cancer, the results obtained in this blind study suggest that the CLIA-CA-62 assay could be a useful tool to supplement existing mammography screening as well as other diagnostic imaging methods, which could improve the diagnostic sensitivity in DCIS and Stage I breast cancer detection thus improving clinical outcomes. Patients having a strong elevation in serum CA-62 level might have another type of carcinoma, which does not make it false positive for BC but rather benefits the simultaneous detection of some other existing cancer.
5. This evaluation of the CLIA-CA-62 chemiluminescent assay for early stages of breast cancer detection strongly suggests it can provide independent and complementary information for the doctors in decision-making and can be considered a useful tool for the primary detection of breast cancer in asymptomatic women. It would be beneficial to use serum CA-62 level in conjunction with the clinical information and other diagnostic procedures.

## Data availability statement

The raw data supporting the conclusions of this article will be made available by the authors, without undue reservation.

## Ethics statement

The studies involving human participants were reviewed and approved by Local Ethics Committee of the Sechenov First Moscow State Medical University. The patients/participants provided their written informed consent to participate in this study.

## Author contributions

Conception, MS. Interpretation or analysis of data, AB, AF, AR, and NB. Preparation of the manuscript, AB, AF, AR, and NB. Revision for important intellectual content, MS and AB. Supervision, MS. All authors contributed to the article and approved the submitted version.

## References

[1] Sung H Ferlay J Siegel RL Laversanne M Soerjomataram I Jemal A. Global cancer statistics 2020: GLOBOCAN estimates of incidence and mortality worldwide for 36 cancers in 185 countries. CA Cancer J Clin (2021) 71(3):209–49. doi: 10.3322/caac.21660 33538338

[2] Lima SM Kehm RD Terry MB. Global breast cancer incidence and mortality trends by region, age-groups, and fertility patterns. EClinicalMedicine (2021) 38:100985. doi: 10.1016/j.eclinm.2021.100985 34278281 PMC8271114

[3] Kong Y-C Bhoo-Pathy N Subramaniam S Bhoo-Pathy N Taib NA Jamaris S. Advanced stage at presentation remains a major factor contributing to breast cancer survival disparity between public and private hospitals in a middle-income country. Int J Environ Res Public Health (2017) 14(4):427. doi: 10.3390/ijerph14040427 28420149 PMC5409628

[4] Rubinstein WS Latimer JJ Sumkin JH Huerbin M Grant SG Vogel VG. Prospective screening study of 0.5 Tesla dedicated magnetic resonance imaging for the detection of breast cancer in young, high-risk women. BMC Women’s Health (2006) 6:10. doi: 10.1186/1472-6874-6-10 16800895 PMC1553433

[5] Peintinger F. National breast screening programs across Europe. Breast Care (2019) 14:354–8. doi: 10.1159/000503715 PMC6940461 31933580

[6] Azadnajafabad S Saeedi Moghaddam S Keykhaei M Shobeiri P Rezaei N Ghasemi E. Expansion of the quality of care index on breast cancer and its risk factors using the global burden of disease study. Cancer Med (2022) 00:1–15. doi: 10.1002/cam4.4951 PMC9883412 35770711

[7] da Costa Vieira RA Biller G Uemura G Ruiz CA Curado MP. Breast cancer screening in developing countries. Clinics (Sao Paulo) (2017) 72(4):244–53. doi: 10.6061/clinics/2017(04)09 PMC5401614 28492725

[8] AJCC cancer staging manual. In Amin MB ed. American College of Surgeons (2017).

[9] Jainish P Prittesh P. Biosensors and biomarkers: promising tools for cancer diagnosis. Int J Biosen Bioelectron (2017) 3(4):313–6. doi: 10.15406/ijbsbe.2017.03.00072

[10] Duffy MJ Walsh S McDermott EW Crown J. Biomarkers in breast cancer: where are we and where are we going? J.Adv Clin Chem (2015) 71:1–23. doi: 10.1016/bs.acc.2015.05.001 26411409

[11] Voronova V Glybochko P Svistunov A Fomin V Kopylov P Tzarkov P. Diagnostic value of combinatorial markers in colorectal carcinoma. Front Oncol (2020) 10:832. doi: 10.3389/fonc.2020.00832 32528895 PMC7258084

[12] Voronova V Peskov K Glybochko P Svistunov A Fomin V Kopylov P. Evaluation and diagnostic potential of plasma biomarkers in bladder cancer. Ann Oncol (2019) 30(5):V45–7. doi: 10.1093/annonc/mdz239

[13] Glybochko PV Svistunov A Fomin V Kopylov P Sekacheva M Vasiliev I. Method for screening probability of breast cancer presence. Patent EA202090713A2 (2020). Available at: https://ru.espacenet.com/publicationDetails/originalDocument?FT=D&date=20210129&DB=EPODOC&locale=ru_RU&CC=EA&NR=202090713A3&KC=A3&ND=4

[14] Bhushan A Gonsalves A Menon JU. Current state of breast cancer diagnosis, treatment, and theranostics. Pharmaceutics (2021) 13:723–30. doi: 10.3390/pharmaceutics13050723 PMC8156889 34069059

[15] Park BW Oh JW Kim JH Park SH Kim KS Kim JH Lee KS. Preoperative CA 15-3 and CEA serum levels as predictor for breast cancer outcomes. Ann Oncol Eur Soc Med Oncol (ESMO) (2008) 19 (4):675–81. doi: 10.1093/annonc/mdm538 18037623

[16] Cherkasova JR Tsurkan SA Kondratiev VB Moro-Vidal R. Cancer antigen for early cancer detection//Patent RU2020114411A, WO2021215955A1 (2021). Available at: https://patents.google.com/patent/WO2021215955A1/en?oq=WO2021215955A1.

[17] Tcherkassova J Prostyakova A Tsurkan S Ragoulin V Boroda A Sekacheva M. Diagnostic efficacy of the new prospective biomarker’s combination CA 15-3 and CA-62 for early-stage breast cancer detection: results of the blind prospective-retrospective clinical study. Cancer Biomarkers (2022) 35(1):57–69. doi: 10.3233/CBM-210533 35786648 PMC12364226

[18] Cherkasova Zh. R Tsurkan SA Kondratiev VB. Set of reagents for detecting the marker for epithelial carcinomas//Patent RU2735918C2, US2022057401A1 (2022). Available at: https://worldwide.espacenet.com/patent/search/family/062981387/publication/US2022057401A1?q=US2022057401A1.

[19] Khakimova GG Cherkasova Z Tsurkan SA Fedchikov GA Suganov NV Gorbunova VA. A pilot clinical trial to monitor response to chemotherapy using the CA-62 marker of epithelial carcinomas. Siberian J Oncol (2019) 18(5):18–28. doi: 10.21294/1814-4861-2019-18-5-18-28

[20] Sawaki M Shien T Iwata H. TNM classification of malignant tumors (Breast cancer study group). Jpn J Clin Oncol (2019) 49(3):228–31. doi: 10.1093/jjco/hyy182 30541035

[21] Leclercq I Batéjat C Burguière AM Manuguerra JC. Heat inactivation of the middle East respiratory syndrome coronavirus. Influenza Other Respir Viruses (2014) 8(5):585–6. doi: 10.1111/irv.12261 PMC4181824 25074677

[22] Tuck MK Chan DW Chia D Godwin AK Grizzle WE Krueger KE. Standard operating procedures for serum and plasma collection: early detection research network consensus statement standard operating procedure integration working group. J Proteome Res (2009) 8(1):113–7. doi: 10.1021/pr800545q PMC2655764 19072545

[23] Duffy MJ. Serum tumor markers in breast cancer: are they of clinical value? Clin Chem (2006) 52(3):345–51. doi: 10.1373/clinchem.2005.059832 16410341

[24] Duffy MJ Shering S Sherry F McDermott E O’Higgins N. CA 15-3: a prognostic marker in breast cancer. Int J Biol Markers (2000) 15(4):330–3. doi: 10.1177/172460080001500410 11192829

[25] Majid AS de Paredes ES Doherty RD Sharma NR Salvador X. Missed breast carcinoma: pitfalls and pearls. Radiographics (2003) 23(4):881–95. doi: 10.1148/rg.234025083 12853663

[26] Giampietro RR Cabral MVG Lima SAM Weber SAT Dos Santos Nunes-Nogueira V. Accuracy and effectiveness of mammography versus mammography and tomosynthesis for population-based breast cancer screening: a systematic review and meta-analysis. Sci Rep (2020) 10 (1):7991. doi: 10.1038/s41598-020-64802-x 32409756 PMC7224282

[27] Hofvind S Geller BM Skelly J Vacek PM. Sensitivity and specificity of mammographic screening as practiced in Vermont and Norway. Br J Radiol (2012) 85(1020):e1226–32. doi: 10.1259/bjr/15168178 PMC3611728 22993383

[28] Cheng R Li J Ji L Liu H Zhu L. Comparison of the diagnostic efficacy between ultrasound elastography and magnetic resonance imaging for breast masses. Exp Ther Med (2018) 15(3):2519–24. doi: 10.3892/etm.2017.5674 PMC5792773 29456656

[29] Morris EA. Rethinking breast cancer screening: ultra FAST breast magnetic resonance imaging. J Clin Oncol (2014) 32(22):2281–3. doi: 10.1200/JCO.2014.56.1514 24958827

[30] Zeeshan M Salam B Khalid QSB Alam S Sayani R. Diagnostic accuracy of digital mammography in the detection of breast cancer. Cureus (2018) 10(4):e2448. doi: 10.7759/cureus.2448 29888152 PMC5991925

[31] Klein EA Richards D Cohn A Tummala M Lapham R Cosgrove D. Clinical validation of a targeted methylation-based multi-cancer early detection test using an independent validation set. Ann Oncol (2021) 32(9):1167–77. doi: 10.1016/j.annonc.2021.05.806 34176681

[32] Gilchrist JM. Weighted 2 x 2 kappa coefficients: recommended indices of diagnostic accuracy for evidence-based practice. J Clin Epidemiol (2009) 62(10):1045–53. doi: 10.1016/j.jclinepi.2008.11.012 19278830

